# Process Analytical Strategies for Size Monitoring: Offline, At-Line, Online, and Inline Methods in a Top-Down Nano-Manufacturing Line

**DOI:** 10.3390/pharmaceutics17060684

**Published:** 2025-05-22

**Authors:** Christina Glader, Ramona Jeitler, Yan Wang, Remy van Tuijn, Albert Grau-Carbonell, Carolin Tetyczka, Steve Mesite, Philippe Caisse, Johannes Khinast, Eva Roblegg

**Affiliations:** 1 Research Center Pharmaceutical Engineering GmbH, Inffeldgasse 13, 8010 Graz, Austria; christina.glader@rcpe.at (C.G.); carolin.tetyczka@rcpe.at (C.T.); johannes.khinast@rcpe.at (J.K.); 2 Institute of Pharmaceutical Sciences, Pharmaceutical Technology & Biopharmacy, University of Graz, Universitätsplatz 1, 8010 Graz, Austria; 3 InProcess-LSP, Kloosterstraat 9, 5349 AB Oss, The Netherlands; y.wang@inprocess-lsp.com (Y.W.); r.vantuijn@inprocess-lsp.com (R.v.T.); a.grau-carbonell@inprocess-lsp.com (A.G.-C.); 4 Microfluidics International Corporation, 90 Glacier Drive, Westwood, MA 02090, USA; smesite@idexcorp.com; 5 Gattefossé SAS, 36 chemin de Genas, 69800 Saint-Priest, France; pcaisse@gattefosse.com; 6 Institute of Process and Particle Engineering, Graz University of Technology, Inffeldgasse 13, 8010 Graz, Austria

**Keywords:** lipid-based nanosystems, real-time size monitoring, continuous top-down manufacturing, PAT tools, dynamic light scattering, spatially resolved dynamic light scattering

## Abstract

**Background/Objectives**: Continuous manufacturing is gaining importance in the nanopharmaceutical field, offering improved process efficiency and product consistency. To fully leverage its potential, the integration of Process Analytical Technology (PAT) tools is essential for real-time quality control and robust process monitoring. Among the critical quality attributes (CQAs) of nanosystems, particle size plays a key role in ensuring product consistency and performance. However, real-time size monitoring remains challenging due to complex process dynamics and nanosystem heterogeneity. **Methods**: This study evaluates the applicability of conventional Dynamic Light Scattering (DLS) and spatially resolved DLS (SR-DLS) using the NanoFlowSizer (NFS) as PAT tools in a temperature-regulated top-down nano-production line. Various lipid-based nanosystems, including solid lipid nanoparticles (SLN), nanostructured lipid carriers (NLC), and nanoemulsions (NEs), were investigated. To ensure reliable implementation, key factors such as sample dilution, viscosity, focus position, measurement angle and temperature effects were systematically assessed for offline and at-line DLS using the Litesizer 500, as well as for offline, inline, and online SR-DLS using the NFS. **Results**: Offline screening confirmed that selecting the appropriate dilution medium and rate ensures measurement reliability. At-line methods provided an efficient alternative by enabling rapid final product control with minimal manual intervention. Inline and online monitoring further enhanced process efficiency by enabling real-time tracking of size, reducing waste, and allowing immediate process adjustments. **Conclusions**: This study demonstrates that integrating offline, at-line, in-line, and online DLS techniques allows for comprehensive product monitoring throughout the entire production line. This approach ensures a streamlined process, enables real-time adjustments, and facilitates reliable quality control after production and during storage.

## 1. Introduction

In the pharmaceutical industry, continuous manufacturing is gaining importance as a strategy to enhance process efficiency, reduce production costs, and ensure consistent product quality [1,2,3]. To fully leverage these benefits, Process Analytical Technology (PAT) tools are essential for real-time monitoring of critical quality attributes (CQAs) ensuring process reliability. This is also valuable in nanopharmaceutical production, where the precise control of CQAs during pharmaceutical manufacturing has garnered increasing attention, leading international regulatory and funding bodies, including the European Union, to prioritize and support research in PAT for nanosystems. Notable examples include EU-funded initiatives such as the NanoPAT and PAT4Nano projects [4]. Among the CQAs, particle size is a key determinant of nanosystem stability, pharmacokinetics, and therapeutic performance, making precise size monitoring essential. Despite progress in nano-size measurement technologies [5,6], real-time monitoring remains a challenge due to the complex dynamics of manufacturing processes and the diverse nature of nanosystems. Therefore, selecting the most appropriate measurement techniques and adapting them to specific process conditions are crucial steps in establishing effective quality control for continuous nanopharmaceutical production. A distinction is made between inline, online, and at-line measurements [7]. Inline monitoring enables direct, real-time size analysis within the process stream, allowing immediate process adjustments. Online measurements involve temporary sample separation but still provide rapid feedback with minimal delay. At-line methods, though requiring sample isolation, offer timely quality control near the production line without significantly impacting process efficiency [8,9,10].

Despite advancements in size characterization, real-time monitoring remains challenging. Conventional dynamic light scattering (DLS) is primarily used offline, requiring process interruptions and leading to delayed feedback and potential batch-to-batch variations, as only a part of the sample is measured. By implementing a pump for sample circulation, a dilution unit, and specific cuvettes into the set-up, it is possible to perform at-line measurements using conventional DLS for final product control [11,12]. Nevertheless, since DLS relies on static measurements, it cannot be directly integrated into the production process for inline or online monitoring of size in flow. Spatially resolved dynamic light scattering (SR-DLS) addresses this limitation by combining DLS with low-coherence interferometry, enabling depth-resolved size analysis and compensating for flow effects—making inline measurements feasible during manufacturing [12].

SR-DLS has been applied in production lines for various nanosystems, including titanium dioxide suspensions, silica nanoparticles, polystyrene beads, lipid-based drug carriers, and emulsions [12,13,14,15,16,17]. However, the complexity and diversity of nanosystems, along with process-specific conditions, pose significant challenges for the successful implementation of SR-DLS. In a recently developed solvent-free top-down manufacturing line for lipid-based nanoparticles, it has been reported that temperature variations influence particle solid-state properties, morphology, and size, which makes reliable inline monitoring difficult [18]. Moreover, the production of highly concentrated samples hinders the Brownian diffusion of particles, complicating the acquisition of reliable measurement data and impeding effective inline size monitoring [19].

Thus, successful integration of at-line, online, and inline size analyzers in nano-manufacturing demands a thorough understanding of measurement conditions and process dynamics. This study evaluated influential factors such as dilution, viscosity, focus position and measurement angle considering the required set-up for offline and at-line measurements based on DLS using the Litesizer 500. SR-DLS using the NanoFlowSizer (NFS) was used for offline, inline and online measurements. To maximize applicability, different lipid-based nanosystems (i.e., solid lipid nanoparticles (SLN), nanostructured lipid carriers (NLC) and nanoemulsions (NEs)) consisting of different lipids (Precirol^®^ ATO 5, Gelucire^®^ 43/01 and Labrafac™ lipophile WL 1349) were investigated. Since SLN and NLC were produced solvent-free via hot homogenization, the influence of different process temperatures and solid states of the nanosystems (i.e., lipid droplets and solid particles) was also taken into account. By combining offline, at-line, inline, and online size monitoring a comprehensive quality control strategy was established that enables continuous product surveillance throughout the entire lifecycle—from manufacturing to final product assessment and storage.

## 2. Materials and Methods

### 2.1. Materials

For the production of lipid-based nanosystems, the solid lipids Precirol^®^ ATO 5 and Gelucire^®^ 43/01 and the liquid lipid Labrafac™ Lipophile WL 1349 were provided by Gattefossé S.A.S. (Saint Priest, France). Tween^®^ 80, which was used as an emulsifier, was obtained from Sigma Aldrich (Munich, Germany). Ultrapurified water (i.e., Milli Q^®^-water (MQ water); TKA MicroPure UV (JWT GmbH, Jena, Germany)) was used for all experiments.

### 2.2. Preparation of Lipid-Based Nanosystems

Three different types of lipid-based nanosystems were prepared:

1. SLN consisting of a solid lipid (i.e., Precirol^®^ ATO 5 (SLN P) and Gelucire^®^ 43/01 (SLN G)),
2. NLC, which additionally comprises a liquid lipid (i.e., Labrafac™ lipophile WL 1349) in a solid-to-liquid lipid ratio of 9:1 (w/w) (i.e., NLC P and NLC G),
3. and nanoemulsions (NEs) consisting only of the liquid lipid Labrafac™ lipophile WL 1349.

For all three types, a constant total lipid amount of 10% (*w*/*w*) dispersed in a 2.5% (*w*/*w*) aqueous Tween^®^ 80 phase was used (see Table 1). SLN and NLC formulations were prepared according to Glader et al. [18]. Briefly, the lipid phase and the aqueous phase were heated separately 10 °C above the melting point of the solid lipids (i.e., process temperature of 55 °C for Gelucire^®^ 43/01 and 70 °C for Precirol^®^ ATO 5 formulations) and mixed using a high shear mixer (Ultra Turrax, IKA-Werke GmbH & Co. KG, Staufen, Germany) applying a speed of 12,000 rpm for 30 s. Next, the obtained hot pre-emulsion was transferred to a thermoregulated Microfluidizer^®^ LM 20 (Microfluidics Inc., Westwood, MA, USA) to homogenize the hot pre-emulsions at matrix-specific process temperatures mentioned earlier [18]. The pressure and cycle number were selected based on previous studies [18] to prepare particles with a defined Z-average of around 150 nm (see Table 1). In the second step, the formulations were automatically transferred to the cooling unit and cooled down to 25 °C and 4 °C, respectively. During the entire process, the temperature was externally controlled via heating and cooling circulators.

The NEs were prepared using the same set-up at ambient temperatures after high shear mixing at 12,000 rpm for 30 s followed by processing with the Microfluidizer^®^ processor (see Table 1).

### 2.3. Characterization Using DLS

**Figure 1:**
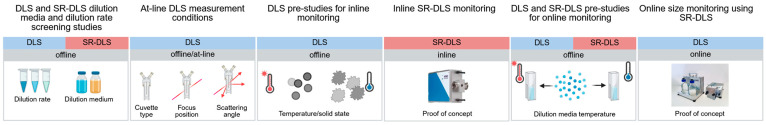
Flowchart summarizing the study design.

DLS measurements were performed using the system from Anton Paar (Litesizer 500, Anton Paar GmbH, Graz, Austria) and data were analyzed via the Anton Paar Kalliope (Anton Paar GmbH, Graz, Austria) software (Version 3.2.4). Size and size distribution are presented as intensity-based Z-average (nm) and polydispersity index (PdI). The refractive index was set to 1.33 for MQ water and Tween^®^ 80 solution, 1.55 for Precirol^®^ ATO 5 formulations, 1.51 for Gelucire^®^ 43/01 formulations and 1.46 for the NEs (see Appendix A—Determination of the RI). If not otherwise stated, measurements were conducted in a disposable cuvette using the back-scatter mode after an equilibration time of 60 s at a temperature of 25 °C (n = 3) (see Figure 1).

### 2.4. Characterization Using SR-DLS

SR-DLS measurements were conducted with the NFS and data were analyzed using the XsperGo 2.1.0.0 software (InProcess-LSP, Oss, The Netherlands). Measurements were performed at a measuring angle of 180° and size distribution was presented as intensity-based cumulant Z-average (nm) and cumulant polydispersity index (PdI) values. Temperature was monitored in real-time and automatically integrated as an input for size characterization (see Figure 1).

### 2.5. DLS and SR-DLS Dilution Media and Dilution Rate Screening Studies

To identify the appropriate dilution media for DLS and SR-DLS size measurements, samples were diluted 1:100 (*v*/*v*) in either MQ water or a 2.5% (*w*/*w*) Tween^®^ 80 solution prior to offline measurements. To further determine the maximum possible particle/droplet concentration, samples were diluted in MQ water with varying dilution factors (i.e., 1:20 to 1:1000 (*v*/*v*)). DLS measurements were performed in a disposable cuvette using the back-scatter mode. SR-DLS measurements were performed in a 10R glass vial with the vial module using the Fides 2 system.

### 2.6. At-Line DLS Measurement Conditions

Different DLS angles (i.e., forward- (15°), side- (90°) and back-scatter (175°)) and different focus positions (i.e., automatic mode and manual focus between −4 and 1 mm) (see Appendix A—Figure A1) were screened offline using disposable (UV-Cuvette semi-micro 759150, Brand GmbH + CO KG, Wertheim, Germany) and the omega cuvettes (mat. no. 225288, Anton Paar GmbH, Graz, Austria) at a 1:100 (*v*/*v*) dilution in MQ water. At-line measurements of sizes were performed through the connection of an automated dosing unit (800 Dosino^®^, Metrohm AG, Herisau, Switzerland) with the omega cuvette using a 1:100 (*v*/*v*) dilution in MQ water (n = 3) (see Figure 1).

### 2.7. DLS Pre-Studies for Inline Monitoring

Temperature-dependent studies were performed using the Univette (Anton Paar GmbH, Graz, Austria) at temperatures representing the process temperature (i.e., 70 °C for Precirol^®^ ATO 5 and 55 °C for Gelucire^®^ 43/01) and the final product temperature (i.e., 25 °C). Given that Gelucire^®^ 43/01 is supposed to be not fully crystalline at 25 °C, additional characterization was conducted at a potential storage temperature of 4 °C. All measurements were conducted offline at a 1:100 (*v*/*v*) dilution in MQ water at the respective temperature (n = 3).

### 2.8. Inline SR-DLS Monitoring

**Figure 2:**
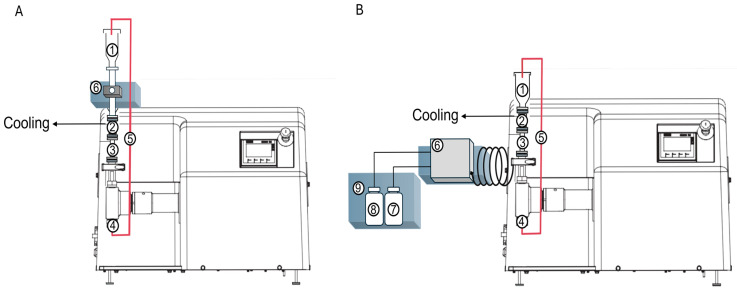
Microfluidizer^®^ processor set-up consisting of the receiver chamber (1), the valve block (2), the feeding chamber (3), the Interaction Chamber™ (4) and the stainless-steel tube (5). The NFS (6) was implemented for inline (**A**) and online (**B**) size characterization, the online set-up additionally comprised the dilution media (7), the product waste (8) and the OMD unit (9).

Inline size monitoring via SR-DLS during Microfluidizer^®^ processing was performed using a 0.25-inch flow cell (InProcess-LSP, Oss, The Netherlands), which was implemented in the Microfluidizer^®^ unit (see Figure 2A). For inline size monitoring, 10 cycles were conducted at the defined pressure (i.e., 500 bar for SLN P and NLC P, 1000 bar for SLN G, NLC G and NE) and samples were measured between the cycles in static conditions at process temperature (n = 5).

### 2.9. DLS and SR-DLS Pre-Studies for Online Monitoring

To study the effect of dilution medium’s temperature on nanosystem size, two strategies were employed during offline DLS screening studies. The first involved the direct dilution of the SLN or NLC formulation at process temperature directly with MQ water pre-conditioned to 25 °C (i.e., hot dilution strategy). In the second strategy, the sample was cooled down to the measurement temperature (i.e., 25 °C) prior to dilution with MQ water (i.e., cold dilution strategy).

For the SR-DLS pre-studies, the hot SLN and NLC samples were separated from the Microfluidizer^®^ process stream after the last cycle, diluted 1:100 (*v*/*v*) in MQ water at room temperature and analyzed offline over time using the vial module. Measurements accounted for the existing temperature, recorded via an integrated PT100 sensor until the samples reached ambient temperature.

### 2.10. Online Size Monitoring Using SR-DLS

For online size monitoring, the Microfluidizer^®^ processor was connected to an Online Micro Dilution (OMD) unit (InProcess-LSP, Oss, The Netherlands) via a short stainless-steel capillary using a smart gasket. This allows controlled cooling of the product to room temperature, similar to the final cooling in the semi-continuous set-up (see Figure 2B). The OMD enables sample dilution via a microgear pump, which was achieved by setting the pump speed to 90% for the sample and 30% for the dispersant (i.e., MQ water), which corresponds to a mass dilution factor of approximately 1:20 (*w*/*w*). For online size monitoring, 5 cycles were conducted at the defined pressure (i.e., 500 bar for SLN P and NLC P, 1000 bar for SLN G, NLC G and NE). Measurements were performed for 5 data points after each cycle at room temperature (see Figure 1).

### 2.11. Statistical Analysis

Unless otherwise stated, experiments were conducted in triplicate and results were presented as mean values ± standard deviation. Statistical analyses were performed via Student’s *t* tests (see Appendix B).

## 3. Results

### 3.1. Offline DLS and SR-DLS Measurement Conditions

#### 3.1.1. Influence of the Dilution Medium on the Measured Size

**Figure 3:**
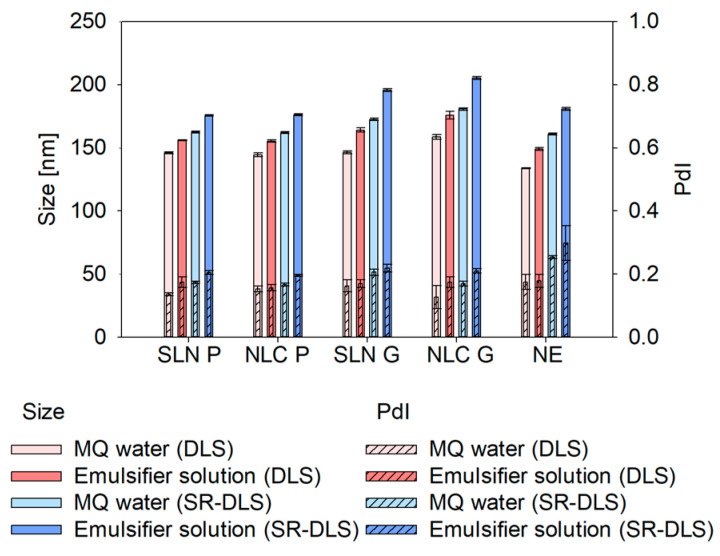
Comparison of DLS and SR-DLS offline measured sizes and PdIs of different SLN, NLC and NE formulations using MQ water and Tween^®^ 80 (emulsifier) solutions as dilution media (dilution rates of 1:100 (*v*/*v*)). Sizes are presented as intensity-based Z-Average (DLS) and cumulant Z-Average (SR-DLS). Statistical data analysis: see Appendix B—Table A3 and Table A4.

The size of all nanoformulations was smaller for samples diluted in MQ water than for those diluted in emulsifier solution (see Figure 3). Differences in size were more pronounced for Gelucire^®^ 43/01 formulations, exhibiting variations of approx. 20–25 nm, compared to Precirol^®^ ATO 5 and NE formulations, which showed variations of approx. 10–15 nm. These deviations were observed in both DLS and SR-DLS measurements. However, SR-DLS measurements showed significantly larger sizes (i.e., about 20–30 nm) compared to DLS (see Appendix B—Table A3). For the PdI, only minor effects of the dilution media and measurement technique were detected (see Appendix B—Table A4). Hence, MQ water was chosen as the dilution medium for the following studies to ensure consistent data and minimize possible effects of the emulsifier on the particle size.

#### 3.1.2. Influence of the Dilution Rate on the Measured Size

**Figure 4:**
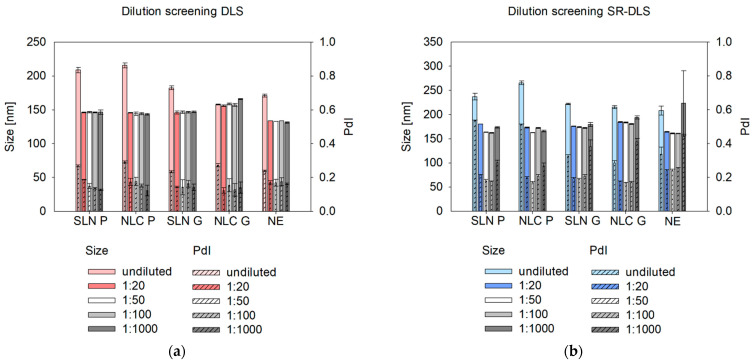
Influence of the dilution rate on the offline DLS (**a**) and SR-DLS (**b**) measured sizes and PdIs of SLN, NLC and NE formulations. Sizes are presented as intensity-based Z-Average (DLS) and cumulant Z-Average (SR-DLS). Statistical data analysis: see Appendix B—Table A5 and Table A6.

Offline size measurements via DLS and SR-DLS showed that regardless of the formulation tested, a dilution factor of at least 1:20 (*v*/*v*) resulted in consistent size and PdI results (see Figure 4). Undiluted nanosystems showed significantly larger sizes independent of the formulation tested (except NLC G) or measurement technique used (see Appendix B—Table A5). Thereby, the size measured via SR-DLS was larger compared to conventional DLS, which coincides with the results of the dilution media screening. To evaluate whether these deviations can be corrected by taking viscosity effects into account, the dynamic viscosity of the individual samples was examined (see Appendix A—Determination of the rheological behavior). The size of the undiluted formulations was recalculated via the software Kalliope (Version 3.2.4) and XSperGo 2 (Version 2.1.0.0) using the sample viscosity instead of the solvent viscosity [6]. However, this approach led to a notable reduction in sizes, except for the NE formulations, which exhibited deviations of less than 10 nm after recalculation (see Appendix A—Figure A3). Accordingly, based on previous results [18] and to enable reliable measurements independently of the formulation, a dilution of 1:100 (*v*/*v*) was selected for further studies.

### 3.2. At-Line DLS Measurement Conditions

**Figure 5:**
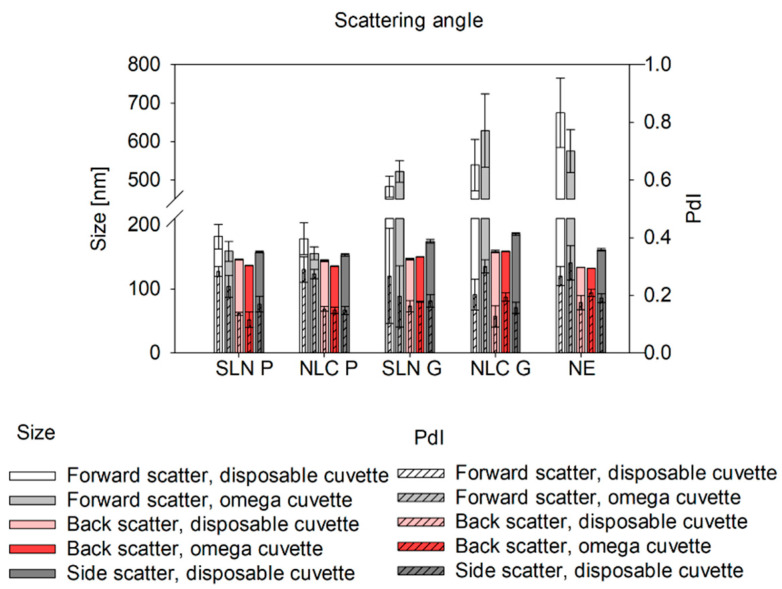
Sizes and PdIs of SLN, NLC and NE formulations at different scattering angles using the disposable and the omega cuvette measured at a 1:100 (*v*/*v*) dilution. Sizes were measured via DLS and are presented as intensity-based Z-Average. Statistical data analysis: see Appendix B—Table A7 and Table A8.

Since at-line measurements with the Litesizer 500 are restricted to omega cuvettes, the comparability of size results between disposable and omega cuvettes was evaluated offline. To assess measurement reliability, screenings for scattering angle and focus position were conducted using both cuvette types. At the forward-scatter angle, no significant size differences were observed between disposable and omega cuvettes. However, size deviations among triplicate measurements ranged from 20 to 70 nm. In contrast, back-scatter measurements showed significant differences between the cuvette types; however, mean size variations remained below 10 nm, with triplicate deviations under 2 nm, indicating robust measurement reliability. Side scatter measurements, which were only possible with disposable cuvettes, yielded significantly larger sizes—approx. 10 nm larger for Precirol^®^ ATO 5 and 30 nm larger for Gelucire^®^ 43/01 and NE formulations—compared to back-scatter measurements (see Figure 5). Regarding PdI, no significant influence of the cuvette type was obtained, except for NLC P. Comparison of the two angles in omega cuvettes (forward- and back-scatter) showed consistently larger sizes and PdI values for forward-scatter measurements. This effect was most pronounced in Gelucire^®^ 43/01 and NE formulations (e.g., differences of 35.6 ± 19.1 nm for SLN P, 34.1 ± 25.1 nm for NLC P, 337.0 ± 27.0 nm for SLN G, 380.6 ± 66.4 nm for NLC G, and 540.6 ± 89.8 nm for NE in the disposable cuvette). Considering these findings and the need for comparability with the NFS, which operates at a fixed 180° scattering angle, the back-scatter angle was selected for subsequent studies to ensure consistent and reliable measurements.

**Figure 6:**
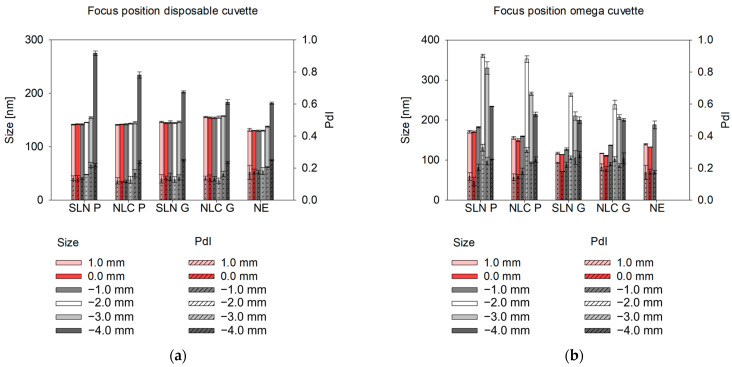
DLS offline measured sizes and PdIs of SLN, NLC and NE at different focus positions (0.0 mm cuvette center, deviations from this center position indicate proximity to the cuvette wall) 1:100 (*v*/*v*) diluted in MQ water using the disposable cuvette (**a**) and the omega cuvette (**b**), respectively. Sizes are presented as intensity-based Z-Average. Statistical data analysis: see Appendix B—Table A9 and Table A10.

Variations in the focus position within the disposable cuvette led to sizes remaining relatively stable between +1 mm and −2 mm, with maximal differences of 4.0 ± 1 nm for SLN P, 2.3 ± 1.4 nm for NLC P, 2.0 ± 1.8 nm for SLN G, 0.4 ± 2.6 nm for NLC G and 1.4 ± 3.0 nm for NE. However, at −3 mm, a slight increase in size was observed, followed by a more pronounced increase at −4 mm. In contrast, in the omega cuvettes, comparable results were only obtained at +1 mm and 0 mm, while significant fluctuations occurred between −1 mm and −4 mm (see Figure 6) (i.e., 11.5 ± 2.5 nm for SLN P, 6.3 ± 1.6 nm for NLC P, 12.3 ± 6.5 nm for SLN G, 24.1 ± 0.8 nm for NLC G and 55.5 ± 9.6 nm for NE). To ensure consistency and comparability between the disposable and omega cuvettes, the focus position was manually set to 0.0 mm, leading to stable and reproducible size measurements.

### 3.3. DLS Pre-Studies for Inline Measurements

**Figure 7:**
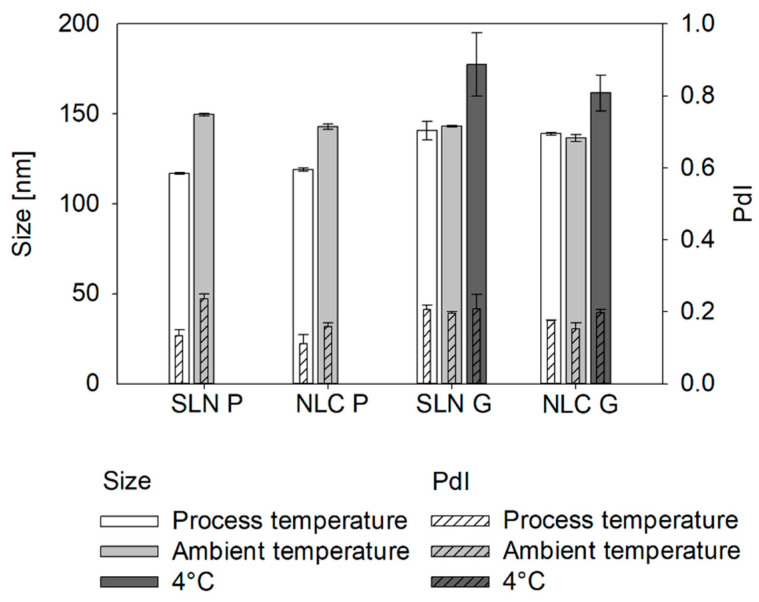
Particle/droplet sizes and PdIs of SLN and NLC formulations offline measured via DLS. At process temperature (white), lipids are in the molten state (i.e., spherical droplets). At ambient temperature (light gray), SLN P and NLC P exist as solid particles (non-perfectly spherical), while SLN G and NLC G start to recrystallize at this temperature (transition from droplets to particles). At 4 °C (dark gray), SLN G and NLC G transitioned to solid particles. Data were assessed offline using DLS and sizes are presented as intensity-based Z-Average. Statistical data analysis: see Appendix B—Table A11 and Table A12.

Due to varying thermal conditions during the processing of SLN and NLC, the nanostructures exist in different physical states during inline measurements (i.e., liquid vs. solid, see Appendix A—Influence of the process temperature on the solid state of SLN and NLC). Different degrees of crystallization can result in variations in size, so measurements were conducted at process temperatures (i.e., 70 °C for Precirol^®^ ATO 5 and 55 °C for Gelucire^®^ 43/01 formulations) and at ambient temperature (i.e., 25 °C). At ambient temperature, Precirol^®^ ATO 5 samples exist in a crystalline state, whereas Gelucire^®^ 43/01 samples are not fully solidified (see Appendix A—Figure A4). To further assess the impact of crystallization on Gelucire^®^ 43/01 samples, additional measurements were conducted at the final storage temperature of 4 °C (see Figure 7). For Precirol^®^ ATO 5 formulations, the sizes measured at process temperature were significantly smaller than those measured at ambient temperature (i.e., a difference of 32.8 ± 1.0 nm for SLN P and 29.9 ± 1.7 nm for NLC P). In contrast, Gelucire^®^ 43/01 formulations showed no significant size differences at this temperature range. However, upon cooling to 4 °C, solidification of the droplets occurred (see Appendix A—Figure A4), leading to a particle size increase of 36.7 ± 18.4 nm for SLN G and 22.7 ± 10.1 nm for NLC G.

### 3.4. DLS and SR-DLS Pre-Studies for Online Measurements

**Figure 8:**
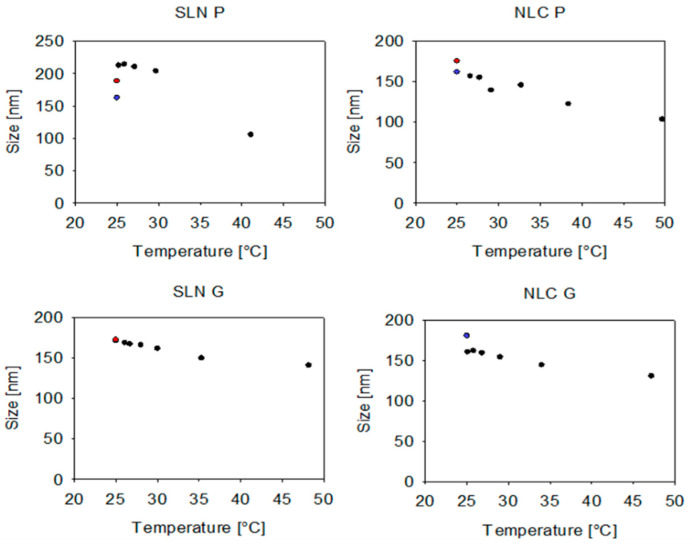
Sizes measured offline using SR-DLS (1:100 diluted with MQ water at ambient temperature) as a function of sample temperature (n = 1) are represented by black dots. DLS-measured particle sizes from the hot dilution strategy are indicated in red, while those from the cold dilution strategy are shown in blue. Note that some data points are not displayed due to overlap, as they correspond to identical particle/droplet sizes (i.e., SLN G and NLC G). However, these data points, along with their corresponding PdI values, can be found in Appendix A—Figure A5. Sizes are presented as intensity-based Z-Average (DLS) and cumulant Z-Average (SR-DLS). Statistical data analysis: see Appendix B—Table A13 and Table A14.

For online measurements using the OMD, sample dilution is performed. For this purpose, different strategies may be pursued. First, in the hot dilution strategy, the hot SLN or NLC formulation is directly diluted with MQ water tempered to 25 °C inducing fast cooling of the droplets via the dilution media. Second, the cold dilution strategy employs cooling the samples to the measurement temperatures (i.e., ambient temperature) before diluting them with MQ water. To investigate the influence of the cooling strategy on the size, both methods were evaluated offline using DLS and SR-DLS. It was found that Precirol^®^ ATO 5 formulations exhibited significantly larger particle sizes and slightly higher PdI values when the hot formulation was diluted directly in MQ water (hot dilution strategy) (i.e., 188.5 ± 1.3 nm and 0.190 ± 0.005 for SLN P, 175.5 ± 1.0 nm and 0.173 ± 0.007 for NLC P), compared to dilution after cooling to 25 °C under controlled conditions (cold dilution strategy) (i.e., 162.4 ± 0.7 nm and 0.174 ± 0.004 for SLN P, 162.0 ± 0.9 nm and 0.167 ± 0.007 for NLC P) (see Figure 8). Conversely, different dilution strategies did not significantly affect the sizes and PdI values of Gelucire^®^ 43/01 formulations (i.e., 172.4 ± 1.0 nm and 0.206 ± 0.010 for SLN G, 180.6 ± 0.8 nm and 0.170 ± 0.007 for NLC G) (see Appendix A—Figure A5). Similar trends were observed in the SR-DLS studies, which showed a significant temperature-dependent variation in size for Precirol^®^ ATO 5 formulations after a 1:100 (*v*/*v*) dilution in MQ water, with smaller sizes noted at elevated temperatures. In contrast, Gelucire^®^ 43/01 formulations exhibited only a minor shift in size when subjected to varying temperatures.

### 3.5. Inline and Online Size Monitoring During Top-Down Nano-Production via SR-DLS

**Figure 9:**
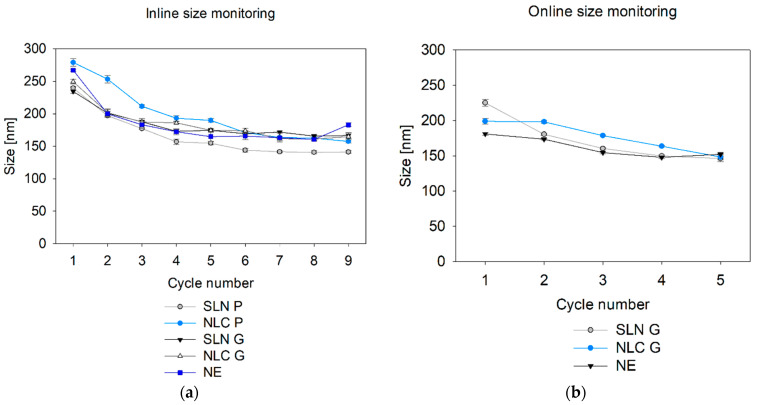
Inline (**a**) and online (**b**) size monitoring after each cycle through the Microfluidizer^®^ processor. Data were assessed via SR-DLS and sizes are presented as cumulant intensity-based Z-Average. Statistical data analysis: see Appendix B—Table A15 and Table A16.

Based on the results of the preliminary studies, real-time inline and online size measurements were conducted by implementing the NFS in the Microfluidizer^®^ set-up. Sizes of different formulations were monitored after each cycle. During inline measurements, a significant reduction in droplet size with an increasing number of cycles was observed regardless of the formulation, before a plateau was reached (see Figure 9a). A similar trend could be observed during online size monitoring of SLN G, NLC G, and NE (see Figure 9b). However, online monitoring of the Precirol^®^ ATO 5 formulations was hindered by the sensitivity of Precirol^®^ ATO 5 particles to temperature changes and uncontrolled cooling conditions, as already indicated in the preliminary studies.

### 3.6. Comparison of Offline, At-Line, Inline, and Online Measured Sizes

**Figure 10:**
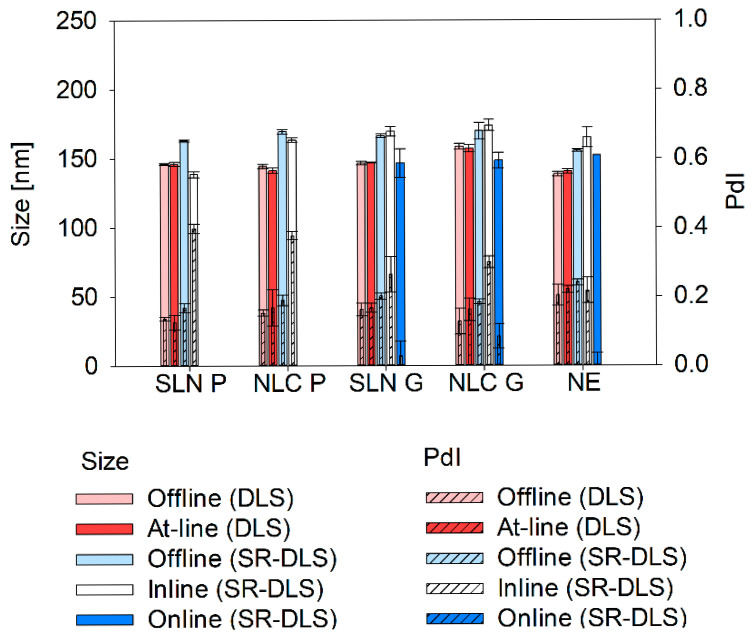
Comparison of sizes determined via offline (DLS and SR-DLS), at-line (DLS), inline (SR-DLS) and online (SR-DLS) measurement strategies. Final products were characterized after 10 cycles at 500 bar for SLN P, 7 cycles at 500 bar for NLC P, 6 cycles at 1000 bar for SLN G, 6 cycles at 1000 bar for NLC G, and 5 cycles at 1000 bar for NE. Inline measurements of droplet sizes were conducted before the cooling step. Offline, at-line, and online measurements were performed after cooling to 25 °C. Sizes of DLS measurements are presented as intensity-based Z-Average and sizes of SR-DLS measurements are presented as cumulant Z-Average values. Statistical data analysis: see Appendix B—Table A17 and Table A18.

After establishing a thorough understanding of the influential measurement parameters for each measurement strategy, differently assessed sizes were compared (see Figure 10). The offline and at-line DLS measurements were not significantly different; the offline SR-DLS data yielded larger sizes, which is in accordance with the preliminary offline screening studies. SR-DLS inline measured sizes of SLN G, NLC G, and NE (i.e., 169.4 ± 3.3 nm, 173.9 ± 4.0 nm, and 164.9 ± 7.2 nm) did not significantly differ (see Appendix B—Table A17) from offline assessed data (i.e., 166.4 ± 1.5 nm, 169.9 ± 6.0 nm, 155.5 ± 0.9 nm) at the pre-defined process conditions (i.e., 1000 bar, 6 cycles for Gelucire^®^ 43/01 formulations and 5 cycles for NE, respectively). In contrast, SLN P and NLC P showed significantly smaller sizes (see Appendix B—Table A17) during the process (i.e., 138.6 ± 2.0 nm and 163.4 ± 1.8 nm) compared to the final product (i.e., 162.9 ± 0.8 nm and 169.5 ± 1.6 nm). Online monitoring revealed significantly smaller sizes (see Appendix B—Table A17) compared to inline and offline measurements (i.e., 146.4 ± 2.3 nm for SLN G, 148.4 ± 6.5 nm for NLC G and 152.2 ± 0.7 nm for NE).

## 4. Discussion

Particle size is widely recognized as a key parameter of nanopharmaceuticals, given its profound influence on the physicochemical stability of formulations and their pharmacokinetic and pharmacodynamic properties. As a result, the implementation of robust and real-time size monitoring systems in continuous nano-manufacturing processes is essential [20,21].

The recently developed SR-DLS shows great potential as a real-time size monitoring technique for continuous nano-manufacturing [12]. By incorporating different modules (i.e., vial module, flow-through cell, and OMD module), it enables offline, inline, and online size measurements. However, obtaining reliable results for each approach requires a thorough understanding of critical measurement parameters and careful consideration of the specific characteristics and limitations of the chosen set-up. Consequently, the successful integration of SR-DLS into inline or online configurations necessitates careful adaptations, often guided by offline data. These adaptations must also account for key sample properties, including nanosystem size, type, dispersion concentration, and turbidity [22]. Alongside the adaptions, it is also recommended to use results from a well-established alternative technique such as DLS as a reference.

In conventional offline DLS as well as at-line DLS and off- and online SR-DLS measurements [23], highly concentrated, turbid nanodispersions must be diluted to ensure adequate scattering intensity for reliable results. Regardless of the technique, selecting a suitable dilution medium is crucial to prevent agglomeration or colloidal changes during measurements. Testing MQ water and the Tween^®^ 80 solution showed that emulsifier-based dilution consistently led to larger sizes across all formulations and measurement strategies (DLS and SR-DLS). This size increase may result from the emulsifier’s tendency to arrange near particle/droplet surfaces, altering hydrodynamic thickness, velocity and consequently the overall hydrodynamic diameter [23]. Additionally, emulsifier adsorption affects particle/droplet-medium interactions, slowing correlation function decay and increasing calculated size [24]. The emulsifier may also form micelles or interact with lipid nanosystems, inducing swelling or fusion, further contributing to size enlargement. While colloidal stability was maintained in both media, MQ water was chosen as the dilution medium for the subsequent studies. Thus, further potential interfering factors between the emulsifier solution and the nanosystems such as the formation of micelles at excess Tween^®^ 80 concentrations or increased interactions at the nanosystem interface could be excluded [23,25].

Apart from the dilution media, also technique-dependent differences were found: sizes measured via SR-DLS were slightly larger compared to conventional DLS. Thereby, differences can be explained as follows: Z-average represents the harmonic mean of the intensity-weighted size distribution. Since scattering intensity depends on the size-to-wavelength ratio, instruments using different wavelengths of light (e.g., 658 nm for the Litersizer 500 vs. 880 nm for the NFS Fides 2) will produce different intensity-weighted size distributions, leading to variations in Z-average and PdI. However, these differences are negligible when all particles are smaller than 0.1λ, as they fall within the Rayleigh scattering regime [15,26].

Beyond selecting a suitable dilution medium, an appropriate dilution rate is essential for offline, at-line, and online measurements to minimize multiple scattering, particle interactions, agglomeration, and electrostatic effects [27]. Multiple scattering can underestimate size in DLS by distorting signals and making nanosystems appear to move faster, leading to a quicker correlation function decay and smaller calculated sizes [28]. SR-DLS, however, mitigates this issue by isolating single scattering signals [11]. In contrast, hindered diffusion at high concentrations can overestimate size, as restricted Brownian motion slows correlation function decay, resulting in larger calculated sizes [29,30]. Based on this study, a minimum dilution of 1:20 (*v*/*v*) is recommended to eliminate multiple scattering and hindered diffusion effects.

To avoid sample dilution, a common strategy described in the literature suggests using the sample viscosity as the input parameter instead of the solvent viscosity to allow for recalculation of true sizes of undiluted samples (see Appendix A—Figure A3) [31,32]. While this approach was applicable to the NE formulation, the SLN and NLC exhibited unrealistic small, recalculated sizes, which can be attributed to the complex rheological behavior of the dispersion. Their non-Newtonian viscosity (see Appendix A—Figure A2) complicates precise viscosity measurements and recalculations [33]. Accordingly, it was found that a dilution rate between 1:20 (*v*/*v*) and 1:100 (*v*/*v*) is suitable for size characterization. Interestingly, at a 1:1000 (*v*/*v*) dilution, SR-DLS reported higher measured sizes and standard deviations, especially for NE samples, which is due to the number of fluctuations that occur when the particle/droplet concentration is too low [34]. These fluctuations affect the scattering signal, leading to an overestimation of size. Therefore, a dilution rate of 1:100 was chosen for further size characterization, which is also consistent with previous protocols [18].

Studies on the comparability of different cuvette types for at-line DLS measurements include screening studies on scattering angle and focus position [27]. In general, the forward-scatter is well-suited for detecting large particles, such as aggregates and agglomerates [35]. In our studies, significantly larger sizes were detected in Gelucire^®^ 43/01 formulations using the forward-scatter compared to back-scatter measurements indicating a small fraction of larger particles. This effect was less pronounced in Precirol^®^ ATO 5 formulations, suggesting a more homogeneous size distribution. Thus, at-line forward-scatter measurements can serve as a tool for final product control to detect even small fractions of larger structures, which can be particularly beneficial in top-down manufacturing strategies. For overall product quality control, however, back-scatter measurements are recommended [36]. They can be used to reduce multiple scattering effects, which enables the analysis of highly concentrated samples [27,34,37]. To further optimize back-scatter angle measurements and extend their applicability, the path length can be altered by changing the focus positions [22,38]. Measurements near the cuvette wall reduce multiple scattering and enable reliable analysis of concentrated, turbid samples [27]. In contrast, measurements in the cuvette center (0.0 mm [38]) are ideal for weakly scattering nanosystems and minimizing hindered diffusion [39], ensuring consistent results for diluted samples. In omega cuvettes used for at-line set-ups, even slight focus shifts (± 1 mm) caused significant size deviations, likely due to their folded capillary geometry, where the cuvette edge is reached faster than in disposable cuvettes. Setting the focus to 0 mm and using back-scatter mode yielded consistent results across cuvette types for offline and at-line measurements. Thus, at-line measurements offer an efficient, automatable alternative for offline final product characterization.

Ideally, product characterization should extend beyond the final product and include monitoring throughout the entire production process. In this context, inline and online measurement strategies are essential components that can be implemented using SR-DLS. As a PAT tool, SR-DLS measurements are performed at a 180° backscatter angle with broadband wavelengths ranging from 850 nm to 910 nm. When using the NFS, the focus position is predefined for each module (i.e., vial module, flow cell module). Any deviations in size vs. depth profile—caused by multiple scattering, high flow rates, or dust particles—are automatically detected and excluded. SR-DLS provides the same measurement parameters and characteristics as conventional DLS, following ISO standards. These include the cumulant Z-Average, an intensity-based harmonic mean particle size, and the PdI, which describes the size distribution. Rapid measurements (~10 s) and inline modules for flows from ~mL/min to over 300 L/h as well as offline measurements allow versatile applications of the instrument: from small-scale laboratory/pilot scale processes to full-scale production pipelines [40].

Novel continuous nano-production lines such as our recently developed top-down production line using the Microfluidizer^®^ technology [18] are operated at elevated temperatures to facilitate the solvent-free production of lipid-based nanosystems. Accordingly, the inline and online monitoring strategies must be capable of providing reliable results even at fluctuating or elevated temperatures, directly affecting the solid state of lipid-based nanosystems.

DSC studies (see Appendix A—Influence of the process temperature on the solid state of SLN and NLC) revealed that at process temperatures, SLN and NLC exist as lipid droplets, which solidify via recrystallization upon cooling and remain solid at storage temperature. Thus, lipid droplets likely convert from a perfect sphere to an irregularly shaped solid particle, increasing the measured hydrodynamic diameter below the recrystallization temperature [41]. This was observed for Precirol^®^ ATO 5 formulations as measured sizes at process temperatures were significantly smaller than at ambient temperatures. Similarly, during OMD pre-studies, the cold dilution maintained particle size whereas the hot dilution resulted in further enlargement, probably due to uncontrolled recrystallization from temperature differences between formulation and dilution medium. This highlights the sensitivity of Precirol^®^ ATO 5 to dilution and cooling, which is crucial for online measurements.

Unlike Precirol^®^ ATO 5, Gelucire^®^ 43/01 formulations, which remain partially liquid at 25 °C, showed no size changes compared to sizes measured at process temperatures. Similarly, hot and cold dilution strategies had no impact on the resulting size. However, cooling to 4 °C led to a significant increase in the measured sizes, indicating a transition from liquid droplets to non-spherical particles.

Finally, inline particle size monitoring during nano-manufacturing provides valuable insights into process progression. This study demonstrated effective real-time tracking of Microfluidizer^®^ processing without requiring sample dilution. For Gelucire^®^ 43/01 formulations and NE, inline measurements after the final Microfluidizer^®^ cycle matched offline sizes after dilution. To date, no studies have explored SR-DLS at elevated temperatures, highlighting the need to understand temperature effects on factors like hindered diffusion in further studies. Inline measurements of Precirol^®^ ATO 5 formulations also captured the size changes observed offline, which were driven by the transition from liquid spherical droplets at process temperature to solid particles at ambient temperature.

If changes in the solid state or sphericity are to be expected, online measurements can be a suitable extension to recapitulate final product properties without massive time delays during the process. By integrating the OMD in the production line, reliable online monitoring of Gelucire^®^ 43/01 and NE formulations during production was achieved with minimal time delay (<1 min). Thereby, the slightly smaller sizes may be attributed to the different modules used. The assumption is that the high curvature of the 0.25-inch flow cell used for online measurements reduces the intensity, causing smaller measured sizes. This needs to be studied in future in more detail. Furthermore, the efficacy of online measurements is influenced by the specific properties of the particles being analyzed. For example, customized cooling and dilution strategies are required for Precirol^®^ ATO 5 formulations to ensure that the measured values align with those of the final product. Consequently, certain modifications to the measurement set-up may be necessary to facilitate online monitoring of these formulations.

## 5. Conclusions

Offline screening of DLS and SR-DLS measurement parameters demonstrated that by careful selection of the appropriate dilution medium and rate, both techniques can provide comparable and reliable results for final product characterization. The use of the vial module in SR-DLS studies further enhances efficiency, enabling direct characterization of the final product within its container in just eight seconds during storage. While offline testing remains essential for verifying accuracy and reliability, at-line measurements provide a more efficient alternative by allowing final product control closer to the production line. Compared to offline DLS, at-line methods offer faster, automated analysis, reducing the need for human intervention. Additionally, at-line DLS can be extended to assess other CQAs, such as surface charge (i.e., zeta potential), further extending its applicability in product monitoring.

Building on the advantages of at-line measurements, inline product monitoring advances continuous manufacturing by enabling real-time characterization during production. This approach prevents potential property changes caused by dilution and minimizes waste by eliminating the need for sample separation. Additionally, real-time detection of deviations combined with a deep process understanding—developed through the design of experiments—allows for timely adjustments to process parameters to achieve the desired product profile. This proactive strategy helps to prevent deviations from being detected only during final product characterization, reducing the risk of batch rejection.

For size monitoring during hot homogenization of lipid-based nanosystems, it is crucial to consider temperature-induced effects on solid-state properties and morphology. In cases where lipids undergo solid-state transitions or require sample dilution, the innovative OMD set-up combined with an appropriate cooling strategy offers a promising solution for online measurements. By replicating final product conditions, the OMD set-up enhances predictive accuracy, potentially reducing processing times as formulations can proceed directly to the next production step upon reaching target specifications.

In summary, this study demonstrates that integrating offline, at-line, inline, and online particle size monitoring—leveraging both conventional and advanced DLS techniques—establishes a comprehensive and holistic size control strategy for nanoproducts, from manufacturing to final product assessment and storage stability, representing a time-efficient solution.

## Figures and Tables

**Table 1:** Matrix composition and process conditions of lipid-based nanosystems.

Matrix Composition						Process Conditions		
	Solid Lipid % (*w*/*w*)	Liquid Lipid % (*w*/*w*)	Emulsifier Tween^®^ 80% (*w*/*w*)		MQ Water % (*w*/*w*)	Pressure [bar]	Cycle Number	Temperature [°C]
SLN P	10	-	2.5		87.5	500	10	70
NLC P	9	1	2.5		87.5	500	7	70
SLN G	10	-	2.5		87.5	1000	6	55
NLC G	9	1	2.5		87.5	1000	6	55
NE	-	10	2.5		87.5	1000	5	Ambient temperature

**Figure A1:**
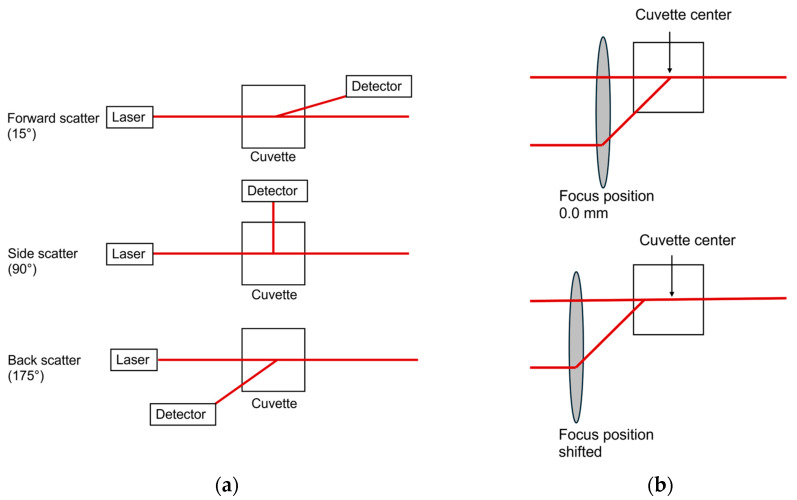
Schematic view of setting the scattering angle (**a**) and focus position (**b**) of DLS measurements using the Litesizer 500, adapted from [42].

**Figure A2:**
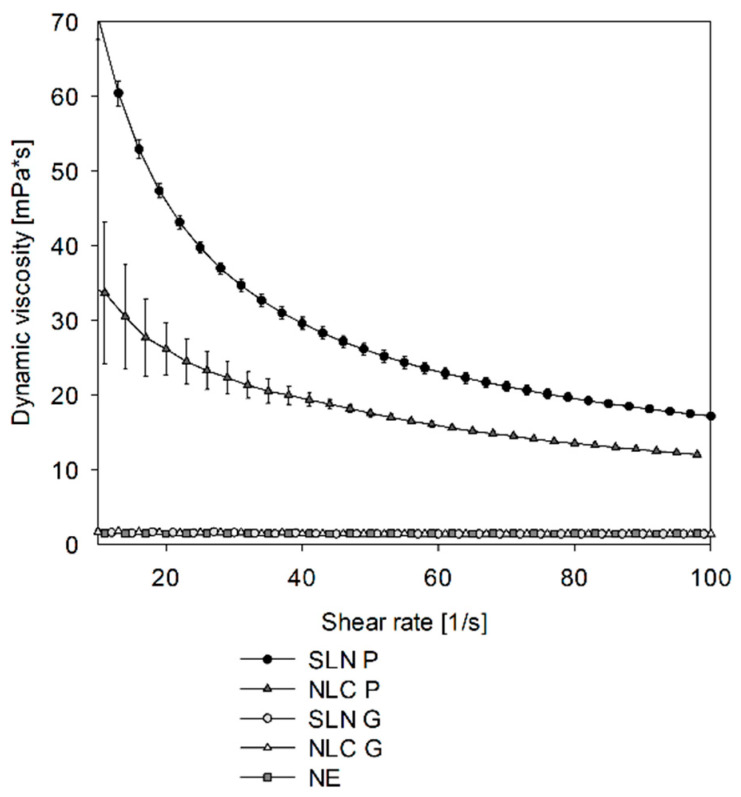
Dynamic viscosity of undiluted SLN, NLC and NE formulations at 22 ± 1 °C.

**Figure A3:**
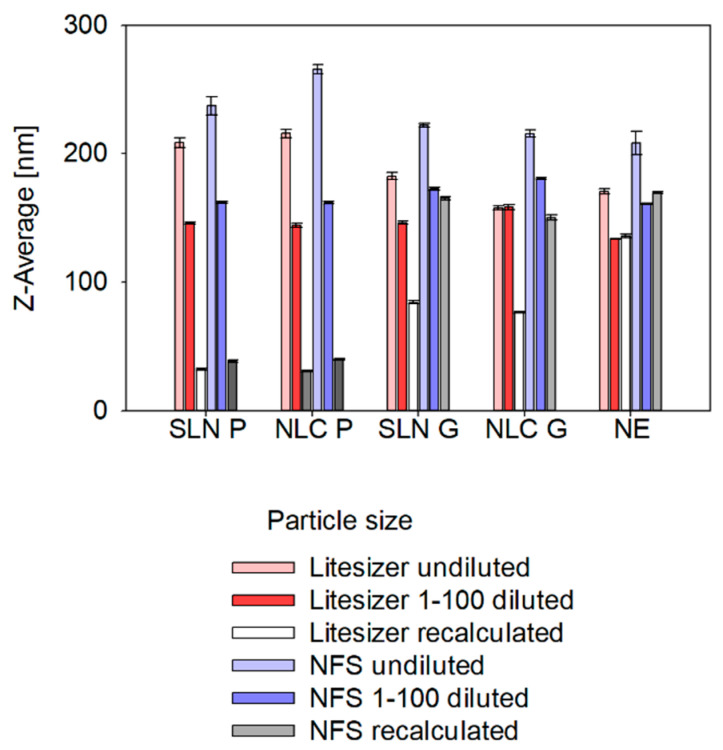
Sizes of different formulations measured with the Litesizer 500 (red, white) and NFS (blue, gray) and recalculated using the sample viscosity as input parameter.

**Figure A4:**
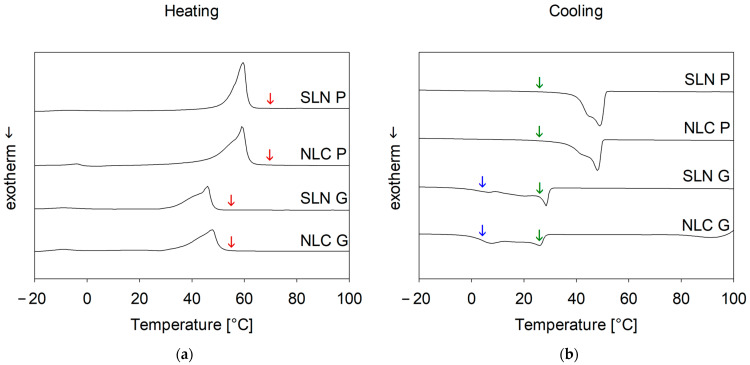
DSC thermograms ((**a**) heating and (**b**) cooling) of SLN P, NLC P, SLN G and NLC G. Red arrows denote the formulation-specific process temperatures (**left**), green arrows represent the measurement temperature at ambient conditions, and blue arrows indicate the measurement temperature of SLN G and NLC G at 4 °C (**right**).

**Figure A5:**
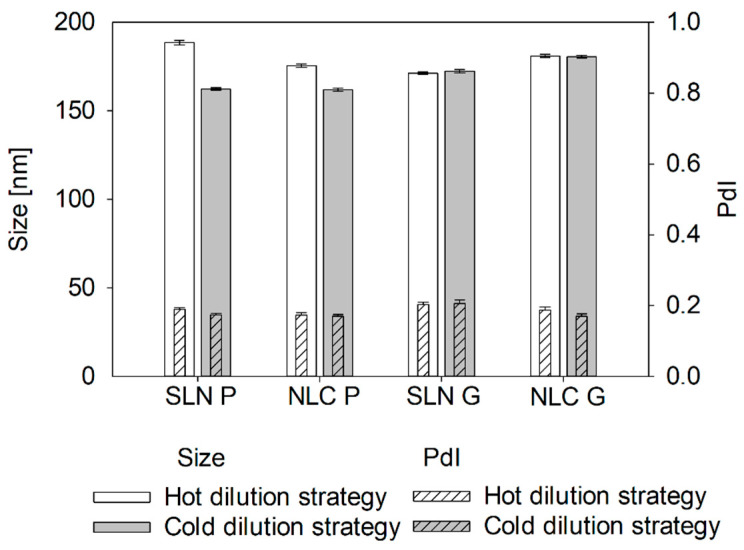
DLS offline measured sizes and PdI values of lipid-based formulations employing two different dilution strategies, i.e., direct dilution of the hot formulation with MQ water tempered to 25 °C (hot dilution strategy) and dilution of samples after controlled cooling to 25 °C (cold dilution strategy) at a dilution rate of 1:100 (*v*/*v*) and at ambient temperatures.

## Data Availability

Data are contained within the article.

## Abbreviations

The following abbreviations are used in this manuscript:

PAT	Process Analytical Technology
CQA	Critical quality attribute
DLS	Dynamic light scattering
SR-DLS	Spatially resolved dynamic light scattering
NFS	NanoFlowSizer
SLN	Solid lipid nanoparticles
NLC	Nanostructured lipid carrier
NE	Nanoemulsion
PdI	Polydispersity index
OMD	Online micro dilution

## Appendix A

### Appendix A.1. Methods

#### Appendix A.1.1. Set-Up of Scattering Angle and Focus Position

For the screening of the scattering angle and focus position in disposable and omega cuvettes, the measurement parameters were adjusted using the Kalliope software according to Figure A1.

#### Appendix A.1.2. Differential Scanning Calorimetry (DSC)

The thermodynamic behavior of SLN and NLC was determined via DSC (204F1 Phoenix, Netzsch GmbH, Selb, Germany). For this purpose, the nanoformulations were air-dried for 72 h and 3–10 mg of the samples were weighed into aluminum crucibles and sealed with a pierced lid. An empty aluminum crucible was used as a reference. The cell was purged at a nitrogen flow rate of 20 mL/min. Samples were scanned between −20 and 100 °C at a heating and cooling rate of 10 °C/min. Measurements were performed in triplicate and data were analyzed using the Netzsch Proteus Software Version 5.2.1 (Netzsch GmbH, Selb, Germany).

#### Appendix A.1.3. Refractive Index (RI)

The refractive index (RI) of lipid-based nanosystems with a 10% (w/w) total lipid amount and their respective dilutions in MQ water (1:2, 1:4, 1:20, 1:50, 1:100, 1:1000, 1:10 000 (*v*/*v*)) were measured using the Abbemat Performance 500 refractometer (Anton Paar GmbH, Graz Austria). In total, 400 μL of the sample dispersion was placed on the glass prism, covered with a lid and illuminated with a LED light of 589 nm at 20 °C. For each nanoparticle formulation (i.e., SLN P, NLC P, SLN G, NLC G, NE), a graph of RI as a function of concentration was plotted and a calibration curve was generated. The RI values of the lipid-based nanosystems (concentration of 100%) were obtained via linear extrapolation.

#### Appendix A.1.4. Viscosity Measurements of Nanodispersions

The dynamic viscosities of SLN and NLC formulations and their respective dilutions with MQ water (1:100 (*v*/*v*)) were investigated with the Physica MCR 301 Rheometer (Anton Paar GmbH, Graz, Austria). Viscosity was measured by applying increasing shear rates ranging from 1 to 100 s^−1^ at 25 °C using a cone-plate geometry (CP-50, radius = 25 mm, cone angle = 0.991°) and a P-PTD200 SN80497110 measuring cell. A »low viscosity« mode was selected and a Peltier system with TruGap™ (Anton Paar GmbH) was used to ensure proper gap size and temperature control. To determine the viscosity of the hot SLN and NLC formulations at process temperatures, samples were isolated from the process stream after passing the Microfluidizer^®^ unit, placed on the pre-tempered Rheometer and characterized at 70 °C (i.e., Precirol^®^ ATO 5) and 55 °C (i.e., Gelucire^®^ 43/01). All measurements were performed in triplicate and the RheoCompass software Version 1.30.1227-Release (Anton Paar GmbH, Graz, Austria) was used for data analysis and regression viscosity calculations.

### Appendix A.2. Results

#### Appendix A.2.1. Determination of the RI

The RI of SLN P, NLC P, SLN G, NLC G and NE and their respective dilutions were measured to obtain the RI values of the lipid-based nanosystems via extrapolation. The correlation coefficient showed high R^2^ values (>0.9993) for all experimental data fitted to the linear function. A calibration curve was created for each formulation and linear equations were established to calculate the RI of the nanosystems:

RI = 0.2279 × c + 1.3326 for SLN P,

RI = 0.2202 × c + 1.3325 for NLC P,

RI = 0.1877 × c + 1.3325 for SLN G,

RI = 0.1757 × c + 1.3324 for NLC G,

RI = 0.1232 × c + 1.3331 for NE,

with c being the concentration (% *w*/*w*) of lipid-based nanosystems. Thus, refractive indices of 1.56, 1.55, 1.52, 1.51 and 1.46 were obtained for SLN P, NLC P, SLN G, NLC G and NE, respectively.

#### Appendix A.2.2. Determination of the Rheological Behavior

Dynamic viscosity measurements at 22 °C revealed shear-thinning properties of the SLN and NLC (see Figure A2), with higher viscosities for Precirol^®^ ATO 5 samples compared to Gelucire^®^ 43/01 (see Table A1). This can be due to the type of lipid but also due to the state of the nanosystems, as Precirol^®^ ATO 5 formulations are fully crystallized, while Gelucire^®^ 43/01 formulations are not completely solidified at this temperature. Furthermore, NLC showed lower viscosities compared to SLN, which may be related to the addition of the liquid lipid [43]. A 1:100 dilution significantly reduced the viscosity for all formulations, resulting in deviations of less than 0.2 mPa·s from that of the dispersant (i.e., MQ water). For size measurements of the diluted formulation, the viscosity of water (i.e., 0.89 mPa·s) was used.

**Table A1:** Regression viscosity of undiluted and 1:100 diluted formulations in [mPa·s] at 22 ± 1 °C.

	Undiluted [mPa·s]	1:100 Diluted [mPa·s]
SLN P	22.55 ± 1.58	1.05 ± 0.02
NLC P	16.15 ± 0.30	1.01 ± 0.01
SLN G	1.62 ± 0.04	0.97 ± 0.04
NLC G	1.46 ± 0.06	0.96 ± 0.01
NE	1.44 ± 0.03	0.90 ± 0.11

Inline size characterization of SLN and NLC is conducted at process temperature, thus the viscosities were additionally investigated at 70 °C and 55 °C, respectively. Table A2 revealed a decrease in viscosity with increasing temperature, consistent with findings reported in the literature [44].

**Table A2:** Regression viscosity of undiluted formulations in mPa·s at process temperature (i.e., 70 °C for Precirol, 55 °C for Gelucire).

	Undiluted [mPa·s]
SLN P	1.29 ± 0.04
NLC P	1.36 ± 0.03
SLN G	0.91 ± 0.01
NLC G	0.98 ± 0.03

#### Appendix A.2.3. Recalculation of the Size Considering the Sample Viscosity

In the measurement of undiluted formulations, the viscosities of the overall formulations were used to recalculate the final size at 22 °C. Except for the NE formulation, recalculations using both Kalliope (Litesizer 500) and XSperGo 2 (NFS) software yielded significantly smaller sizes (see Figure A3). This discrepancy may be attributed to the complex viscosity characteristics of SLN and NLC, as well as additional factors not accounted for in this recalculation, such as multiple scattering effects and hindered diffusion.

#### Appendix A.2.4. Influence of the Process Temperature on the Solid State of SLN and NLC

The thermal behavior of the bulk lipids and physical mixtures was previously investigated to set the process temperatures (i.e., 10 °C above the melting point of the bulk solid lipids) [18]. To evaluate the solid state of the nanosystems at different process conditions and thus different temperatures, DSC measurements were performed with the air-dried SLN and NLC. SLN P showed a melting peak at 60.1 ± 0.3 °C (onset at 57.0 ± 0.1 °C), which was comparable to the bulk Precirol^®^ ATO 5. The melting peak of NLC P shifted to 59.4 ± 0.3 °C (onset of 55.4 ± 0.1 °C) and an additional peak at −4.4 °C was observed, which can be attributed to the liquid lipid Labrafac™ lipophile WL 1349. During the cooling cycles, SLN P showed a crystallization peak at 50.0 ± 0.1 °C (end at 52.3 ± 0.1 °C) and NLC P at 48.4 ± 0.1 °C (end at 51.0 ± 0.1 °C). Consequently, at a process temperature of 70 °C, SLN P and NLC P are present as liquid droplets, whereas at ambient temperature, the Precirol formulations are likely to be solidified. The DSC studies of the Gelucire^®^ 43/01 formulations showed an endothermic event of SLN G and NLC G at 46.2 ± 0.1 °C (onset at 39.3 ± 1.7 °C) and 47.1 ± 0.8 °C (onset at 39.1 ± 3.1 °C), respectively, during heating indicating the melting of the solid lipid. The cooling curves showed a broad exothermic event signifying crystallization ranging between −2.2 ± 1.0 °C and 29.8 ± 0.1 °C for SLN G and −0.6 ± 0.2 °C and 28.4 ± 0.2 °C for NLC G (see Figure A4), respectively. Accordingly, SLN G and NLC G exist as molten lipid droplets at a process temperature of 55 °C but are not fully solid at ambient temperature. However, they are expected to be at least partially solid at 4 °C.

#### Appendix A.2.5. Influence of the Sample Dilution Strategy on the Measured Sizes

Based on the results displayed in Figure 8, Figure A5 provides detailed information regarding particle size and PdI values of the different dilution strategies measured offline via DLS.

## Appendix B

### Statistical Analysis

**Table A3:** Statistical analysis of the sizes of the dilution medium screening (i.e., Figure 3).

		SLN P	NLC P	SLN G	NLC G	NE
MQ water vs. Stab.	DLS	*p* = <0.001	*p* = <0.001	*p* = <0.001	*p* = 0.001	*p* = <0.001
	SR-DLS	*p* = <0.001	*p* = <0.001	*p* = <0.001	*p* = <0.001	*p* = 0.239
DLS vs. SR-DLS	MQ water	*p* = <0.001	*p* = <0.001	*p* = <0.001	*p* = <0.001	*p* = <0.001
	Stabilizer	*p* = <0.001	*p* = <0.001	*p* = <0.001	*p* = <0.001	*p* = 0.121

**Table A4:** Statistical analysis of the PdI of the dilution medium screening (i.e., Figure 3).

		SLN P	NLC P	SLN G	NLC G	NE
MQ water vs. Stab.	DLS	*p* = 0.022	*p* = 0.678	*p* = 0.633	*p* = 0.116	*p* = 0.834
	SR-DLS	*p* = 0.002	*p* = <0.001	*p* = 0.227	*p* = 0.002	*p* = 0.236
DLS vs. SR-DLS	MQ water	*p* = <0.001	*p* = 0.071	*p* = 0.024	*p* = 0.123	*p* = 0.005
	Stabilizer	*p* = 0.046	*p* = 0.003	*p* = 0.008	*p* = 0.031	*p* = 0.025

**Table A5:** Statistical analysis of the sizes of the dilution screening (i.e., Figure 4).

		SLN P	NLC P	SLN G	NLC G	NE
DLS	undiluted vs. 1:100	*p* = <0.001	*p* = <0.001	*p* = <0.001	*p* = 0.621	*p* = <0.001
	1:20 vs. 1:100	*p* = 0.959	*p* = 0.238	*p* = 0.788	*p* = 0.458	*p* = 0.896
	1:50 vs. 1:100	*p* = 0.602	*p* = 0.968	*p* = 0.926	*p* = 0.388	*p* = 0.002
	1:1000 vs. 1:100	*p* = 0.842	*p* = 0.401	*p* = 0.542	*p* = 0.002	*p* = 0.009
SR-DLS	undiluted vs. 1:100	*p* = <0.001	*p* = <0.001	*p* = <0.001	*p* = <0.001	*p* = <0.001
	1:20 vs. 1:100	*p* = <0.001	*p* = 0.488	*p* = 0.005	*p* = 0.007	*p* = 0.004
	1:50 vs. 1:100	*p* = 0.035	*p* = <0.001	*p* = 0.113	*p* = 0.033	*p* = 0.925
	1:1000 vs. 1:100	*p* = <0.001	*p* = 0.003	*p* = 0.037	*p* = 0.003	*p* = 0.182
DLS vs. SR-DLS	undiluted	*p* = 0.004	*p* = <0.001	*p* = <0.001	*p* = <0.001	*p* = 0.002
	1:20	*p* = <0.001	*p* = <0.001	*p* = <0.001	*p* = <0.001	*p* = <0.001
	1:50	*p* = <0.001	*p* = <0.001	*p* = <0.001	*p* = <0.001	*p* = <0.001
	1:100	*p* = <0.001	*p* = <0.001	*p* = <0.001	*p* = <0.001	*p* = <0.001
	1:1000	*p* = <0.001	*p* = <0.001	*p* = <0.001	*p* = <0.001	*p* = 0.076

**Table A6:** Statistical analysis of the PdI of the dilution screening (i.e., Figure 4).

		SLN P	NLC P	SLN G	NLC G	NE
DLS	undiluted vs. 1:100	*p* = <0.001	*p* = <0.001	*p* = 0.003	*p* = 0.003	*p* = 0.011
	1:20 vs. 1:100	*p* = <0.001	*p* = 0.199	*p* = 0.155	*p* = 0.852	*p* = 0.826
	1:50 vs. 1:100	*p* = 0.271	*p* = 0.193	*p* = 0.508	*p* = 0.429	*p* = 0.765
	1:1000 vs. 1:100	*p* = 0.071	*p* = 0.180	*p* = 0.266	*p* = 0.672	*p* = 0.410
SR-DLS	undiluted vs. 1:100	*p* = <0.001	*p* = <0.001	*p* = <0.001	*p* = <0.001	*p* = 0.027
	1:20 vs. 1:100	*p* = <0.001	*p* = 0.275	*p* = 0.116	*p* = 0.381	*p* = 0.046
	1:50 vs. 1:100	*p* = 0.551	*p* = 0.005	*p* = 0.055	*p* = 0.451	*p* = 0.065
	1:1000 vs. 1:100	*p* = <0.001	*p* = 0.008	*p* = 0.002	*p* = <0.001	*p* = <0.001
DLS vs. SR-DLS	undiluted	*p* = <0.001	*p* = <0.001	*p* = <0.001	*p* = 0.260	*p* = 0.016
	1:20	*p* = <0.001	*p* = 0.124	*p* = <0.001	*p* = 0.005	*p* = <0.001
	1:50	*p* = 0.048	*p* = 0.647	*p* = 0.156	*p* = 0.651	*p* = 0.005
	1:100	*p* = <0.001	*p* = 0.002	*p* = 0.024	*p* = 0.123	*p* = 0.005
	1:1000	*p* = <0.001	*p* = 0.002	*p* = <0.001	*p* = <0.001	*p* = <0.001

**Table A7:** Statistical analysis of the sizes of the scattering angle studies (i.e., Figure 5).

		SLN P	NLC P	SLN G	NLC G	NE
Disposable vs. Omega	Forward	*p* = 0.184	*p* = 0.216	*p* = 0.159	*p* = 0.254	*p* = 0.180
	Backscatter	*p* = <0.001	*p* = <0.001	*p* = 0.009	*p* = 0.988	*p* = 0.001
Forward vs. Backscatter	disposable	*p* = 0.032	*p* = 0.078	*p* = <0.001	*p* = <0.001	*p* = <0.001
	omega	*p* = 0.069	*p* = 0.025	*p* = <0.001	*p* = 0.001	*p* = <0.001
Side vs. Backscatter	disposable	*p* = <0.001	*p* = 0.003	*p* = <0.001	*p* = <0.001	*p* = <0.001

**Table A8:** Statistical analysis of the PdI of the scattering angle studies (i.e., Figure 5).

		SLN P	NLC P	SLN G	NLC G	NE
Disposable vs. Omega	Forward	*p* = 0.097	*p* = 0.589	*p* = 0.565	*p* = 0.043	*p* = 0.309
	Backscatter	*p* = 0.261	*p* = 0.574	*p* = 0.230	*p* = 0.043	*p* = 0.101
Forward vs. Backscatter	disposable	*p* = <0.001	*p* = 0.006	*p* = 0.337	*p* = 0.117	*p* = 0.017
	omega	*p* = 0.013	*p* = <0.001	*p* = 0.784	*p* = 0.003	*p* = 0.041
Side vs. Backscatter	disposable	*p* = 0.110	*p* = 0.615	*p* = 0.336	*p* = 0.288	*p* = 0.391

**Table A9:** Statistical analysis of the size of the focus position screening (i.e., Figure 6) compared to the focus position 0.0 mm.

		SLN P	NLC P	SLN G	NLC G	NE
Disposable	1 mm	*p* = 0.214	*p* = 0.189	*p* = 0.108	*p* = 0.393	*p* = 0.490
	−1 mm	*p* = 0.592	*p* = 0.858	*p* = 0.340	*p* = 0.597	*p* = 0.774
	−2 mm	*p* = 0.005	*p* = 0.102	*p* = 0.906	*p* = 0.673	*p* = 0.877
	−3 mm	*p* = <0.001	*p* = 0.081	*p* = 0.135	*p* = 0.021	*p* = <0.001
	−4 mm	*p* = <0.001	*p* = <0.001	*p* = <0.001	*p* = <0.001	*p* = <0.001
Disposable	1 mm	*p* = 0.023	*p* = 0.373	*p* = 0.002	*p* = <0.001	*p* = 0.002
	−1 mm	*p* = 0.001	*p* = 0.002	*p* = 0.004	*p* = <0.001	*p* = <0.001
	−2 mm	*p* = <0.001	*p* = <0.001	*p* = <0.001	*p* = <0.001	-
	−3 mm	*p* = <0.001	*p* = <0.001	*p* = <0.001	*p* = <0.001	-
	−4 mm	*p* = <0.001	*p* = <0.001	*p* = <0.001	*p* = <0.001	-

**Table A10:** Statistical analysis of the PdI of the focus position screening (i.e., Figure 6) compared to the focus position 0.0 mm.

		SLN P	NLC P	SLN G	NLC G	NE
Disposable	1 mm	*p* = 0.972	*p* = 0.631	*p* = 0.744	*p* = 0.893	*p* = 0.859
	−1 mm	*p* = 0.860	*p* = 0.594	*p* = 0.663	*p* = 0.863	*p* = 0.744
	−2 mm	*p* = 0.088	*p* = 0.404	*p* = 0.454	*p* = 0.399	*p* = 0.824
	−3 mm	*p* = 0.004	*p* = 0.017	*p* = 0.772	*p* = 0.154	*p* = 0.036
	−4 mm	*p* = 0.002	*p* = <0.001	*p* = <0.001	*p* = 0.001	*p* = 0.001
Disposable	1 mm	*p* = 0.202	*p* = 0.711	*p* = <0.001	*p* = 0.524	*p* = 0.013
	−1 mm	*p* = 0.011	*p* = 0.077	*p* = 0.009	*p* = 0.036	*p* = 0.014
	−2 mm	*p* = <0.001	*p* = <0.001	*p* = <0.001	*p* = 0.006	-
	−3 mm	*p* = 0.003	*p* = <0.001	*p* = 0.025	*p* = 0.134	-
	−4 mm	*p* = <0.001	*p* = <0.001	*p* = <0.001	*p* = 0.037	-

**Table A11:** Statistical analysis of the sizes of the temperature and cooling condition screening (i.e., Figure 7).

	SLN P	NLC P	SLN G	NLC G
Process vs. ambient temperature	*p* = <0.001	*p* = <0.001	*p* = 0.484	*p* = 0.123
Ambient temperature vs. 4 °C	-	-	*p* = 0.026	*p* = 0.018
Process temperature vs. 4 °C	-	-	*p* = 0.179	*p* = 0.591

**Table A12:** Statistical analysis of the PdI of the temperature and cooling condition screening (i.e., Figure 7).

	SLN P	NLC P	SLN G	NLC G
Process vs. ambient temperature	*p* = 0.016	*p* = 0.263	*p* = 0.008	*p* = 0.085
Ambient temperature vs. 4 °C	-	-	*p* = 0.338	*p* = 0.012
Process temperature vs. 4 °C	-	-	*p* = 0.654	*p* = 0.050

**Table A13:** Statistical analysis of the size of the dilution strategy screening (i.e., Figure 8).

	SLN P	NLC P	SLN G	NLC G
Hot vs. cold dilution strategy	*p* = <0.001	*p* = <0.001	*p* = 0.179	*p* = 0.591

**Table A14:** Statistical analysis of the PdI of the dilution strategy screening (i.e., Figure 8).

	SLN P	NLC P	SLN G	NLC G
Hot vs. cold dilution strategy	*p* = 0.016	*p* = 0.263	*p* = 0.654	*p* = 0.050

**Table A15:** Statistical analysis of inline measured sizes during the Microfluidizer^®^ processing (i.e., Figure 9a).

	SLN P	NLC P	SLN G	NLC G	NE
1 vs. 2 cycle	*p* = <0.001	*p* = 0.006	*p* = <0.001	*p* = <0.001	*p* = <0.001
2 vs. 3 cycle	*p* = <0.001	*p* = <0.001	*p* = 0.022	*p* = 0.029	*p* = 0.010
3 vs. 4 cycle	*p* = 0.001	*p* = 0.002	*p* = 0.003	*p* = 0.659	*p* = 0.056
4 vs. 5 cycle	*p* = 0.478	*p* = 0.327	*p* = 0.393	*p* = 0.005	*p* = 0.209
5 vs. 6 cycle	*p* = 0.007	*p* = 0.002	*p* = 0.066	*p* = 0.832	*p* = 0.893
6 vs. 7 cycle	*p* = 0.248	*p* = 0.020	*p* = 0.343	*p* = 0.015	*p* = 0.661
7 vs. 8 cycle	*p* = 0.671	*p* = 0.481	*p* = 0.038	*p* = 0.766	*p* = 0.529
8 vs. 9 cycle	*p* = 0.795	*p* = 0.029	*p* = 0.778	*p* = 0.296	*p* = <0.001
1 vs. last cycle	*p* = <0.001	*p* = <0.001	*p* = <0.001	*p* = <0.001	*p* = <0.001

**Table A16:** Statistical analysis of online measured sizes during the Microfluidizer^®^ processing (see Figure 9b).

	SLN P	NLC P	SLN G	NLC G	NE
1 vs. 2 cycle	-	-	*p* = <0.001	*p* = 0.769	*p* = 0.005
2 vs. 3 cycle	-	-	*p* = <0.001	*p* = <0.001	*p* = <0.001
3 vs. 4 cycle	-	-	*p* = <0.001	*p* = <0.001	*p* = 0.008
4 vs. 5 cycle	-	-	*p* = 0.097	*p* = 0.016	*p* = 0.012
1 vs. last cycle	-	-	*p* = <0.001	*p* = <0.001	*p* = <0.001

**Table A17:** Statistical analysis of the size determined via different measurement strategies (i.e., Figure 10).

	SLN P	NLC P	SLN G	NLC G	NE
Offline (Litesizer) vs. at-line (Litesizer)	*p* = 0.938	*p* = 0.094	*p* = 0.492	*p* = 0.461	*p* = 0.255
Offline (Litesizer) vs. Offline (NFS)	*p* = <0.001	*p* = <0.001	*p* = <0.001	*p* = 0.010	*p* = <0.001
Offline (NFS) vs. inline (NFS	*p* = <0.001	*p* = <0.001	*p* = 0.169	*p* = 0.194	*p* = 0.051
Offline (NFS) vs. online (NFS)	-	-	*p* = <0.001	*p* = <0.001	*p* = <0.001
Inline (NFS) vs. online (NFS)	-	-	*p* = <0.001	*p* = <0.001	*p* = <0.001

**Table A18:** Statistical analysis of the PdI determined via different measurement strategies (i.e., Figure 10).

	SLN P	NLC P	SLN G	NLC G	NE
Offline (Litesizer) vs. at-line (Litesizer)	*p* = 0.417	*p* = 0.641	*p* = 0.726	*p* = 0.286	*p* = 0.408
Offline (Litesizer) vs. Offline (NFS)	*p* = 0.016	*p* = 0.002	*p* = <0.001	*p* = <0.001	*p* = 0.003
Offline (NFS) vs. inline (NFS	*p* = <0.001	*p* = <0.001	*p* = 0.001	*p* = <0.001	*p* = 0.066
Offline (NFS) vs. online (NFS)	-	-	*p* = <0.001	*p* = <0.001	*p* = 0.874
Inline (NFS) vs. online (NFS)	-	-	*p* = <0.001	*p* = <0.001	*p* = 0.133
